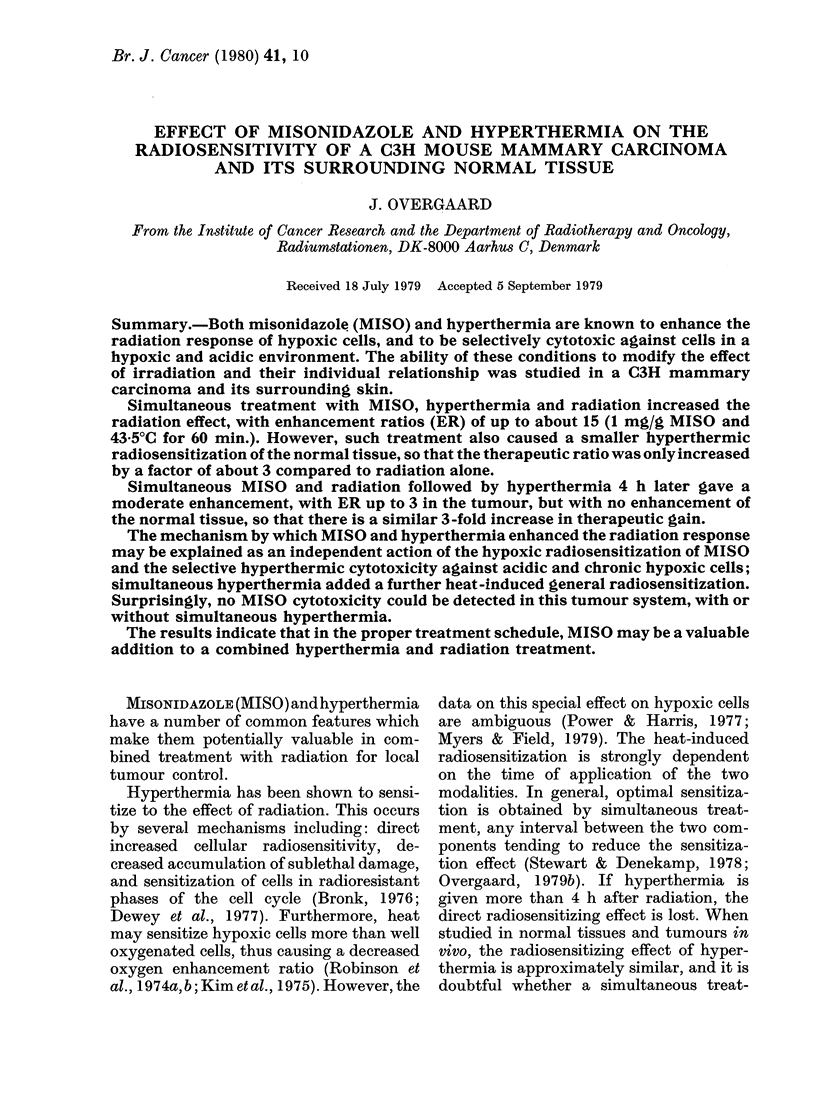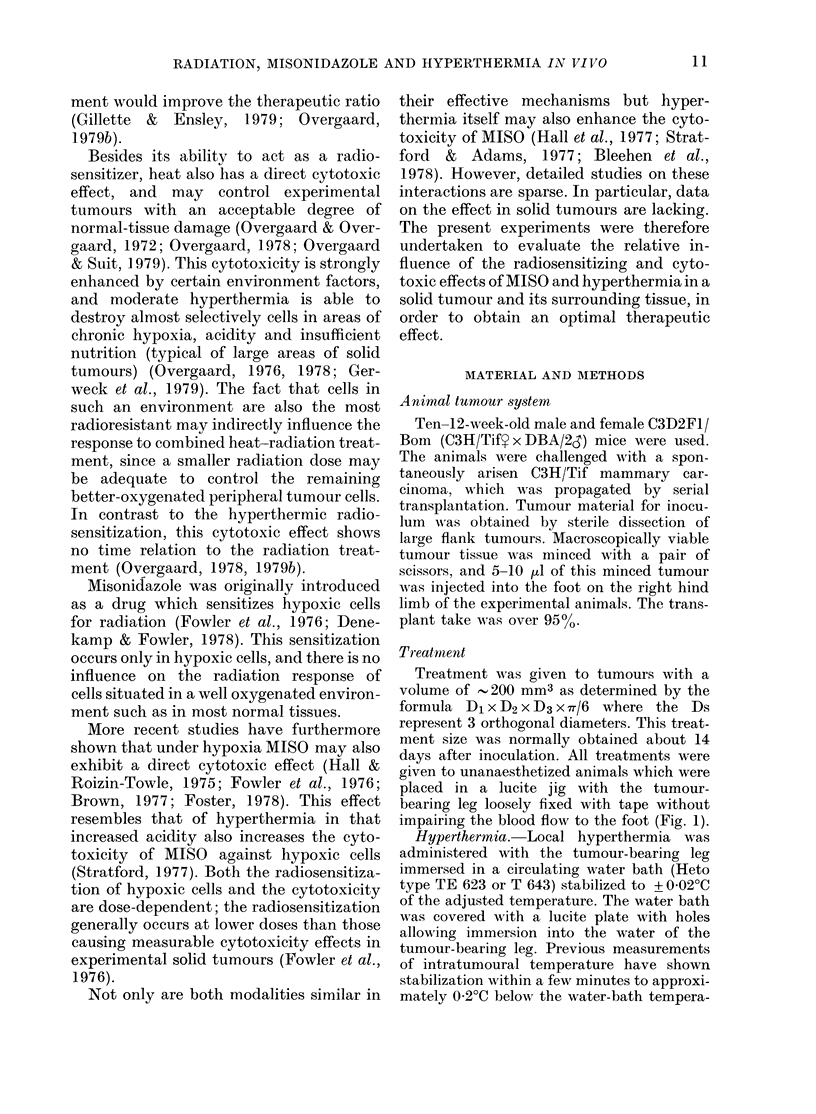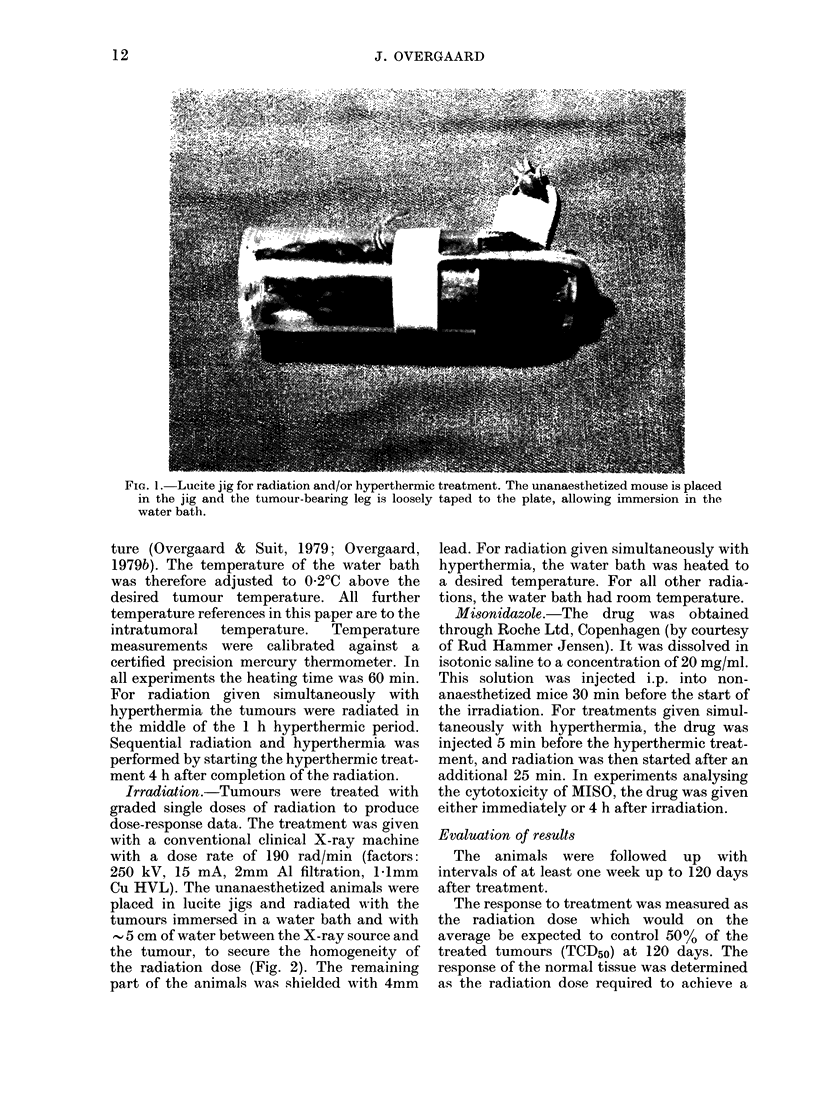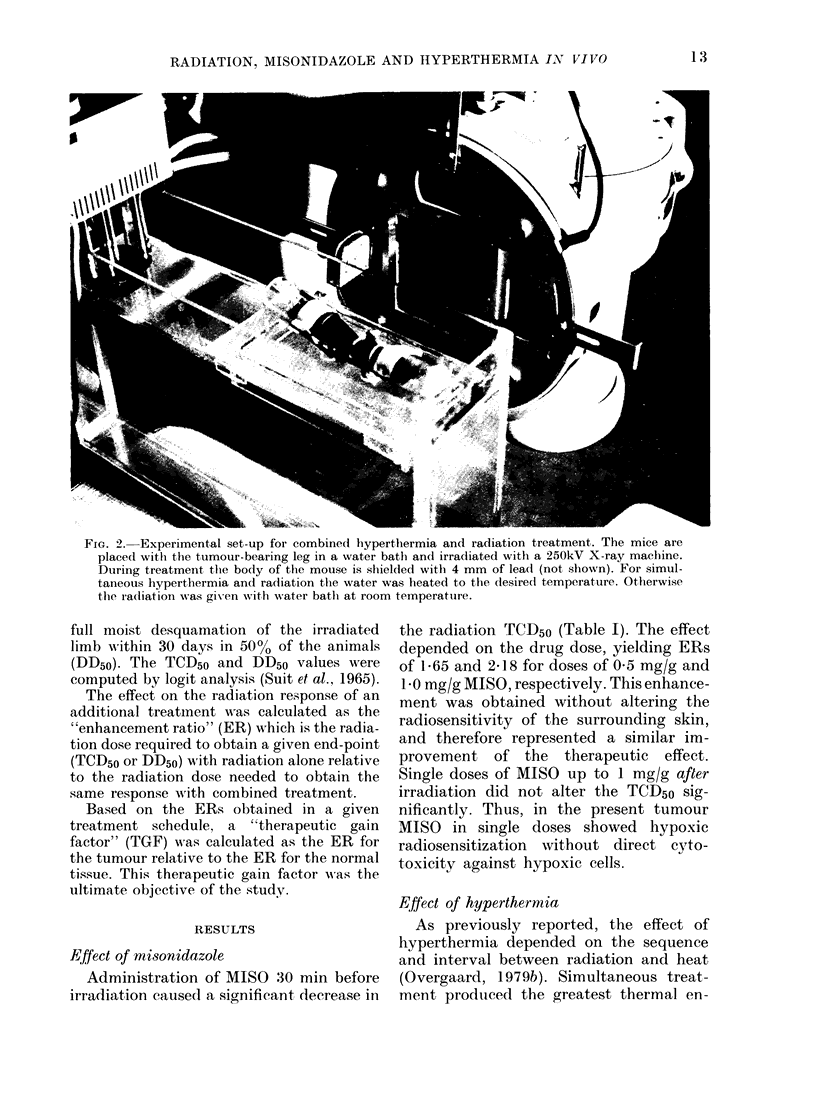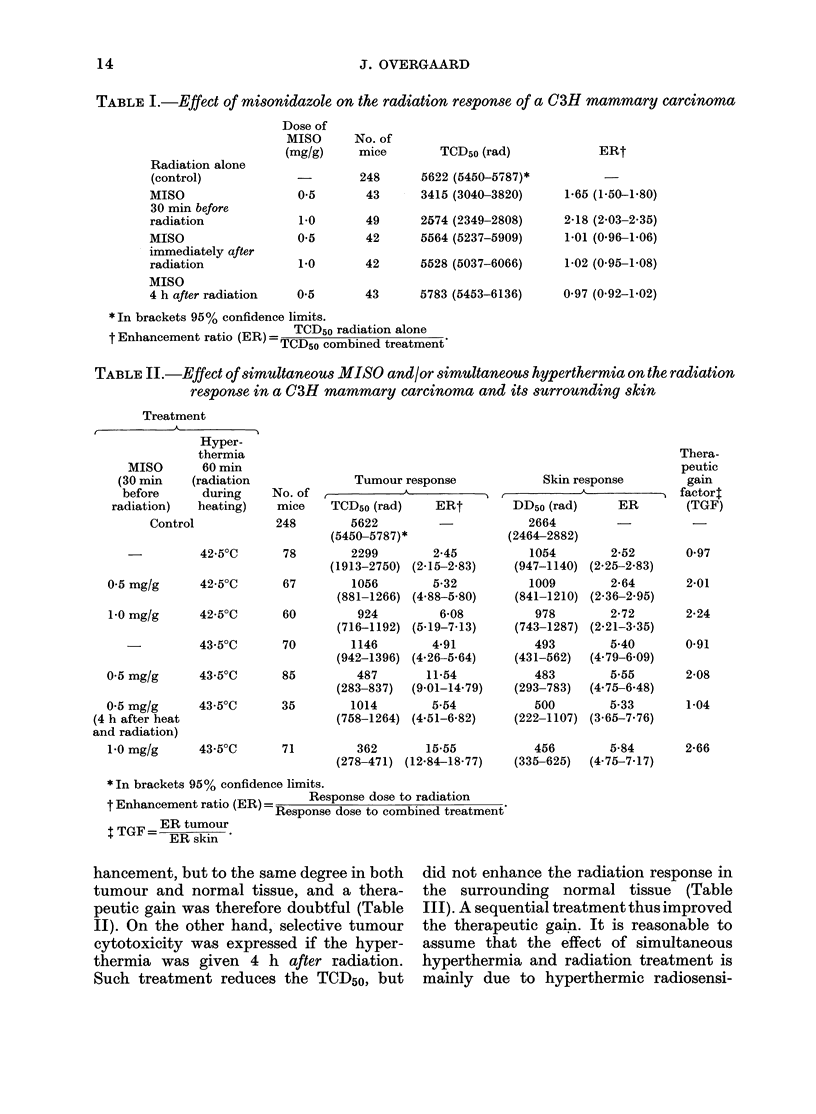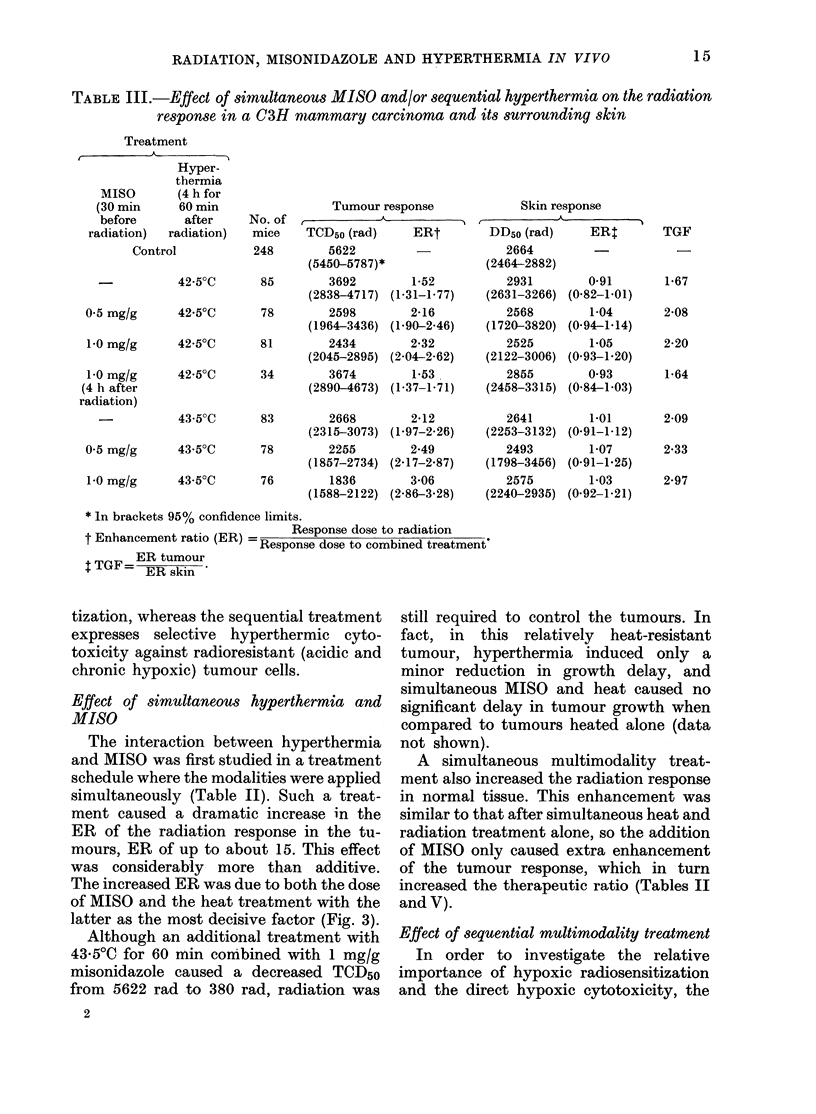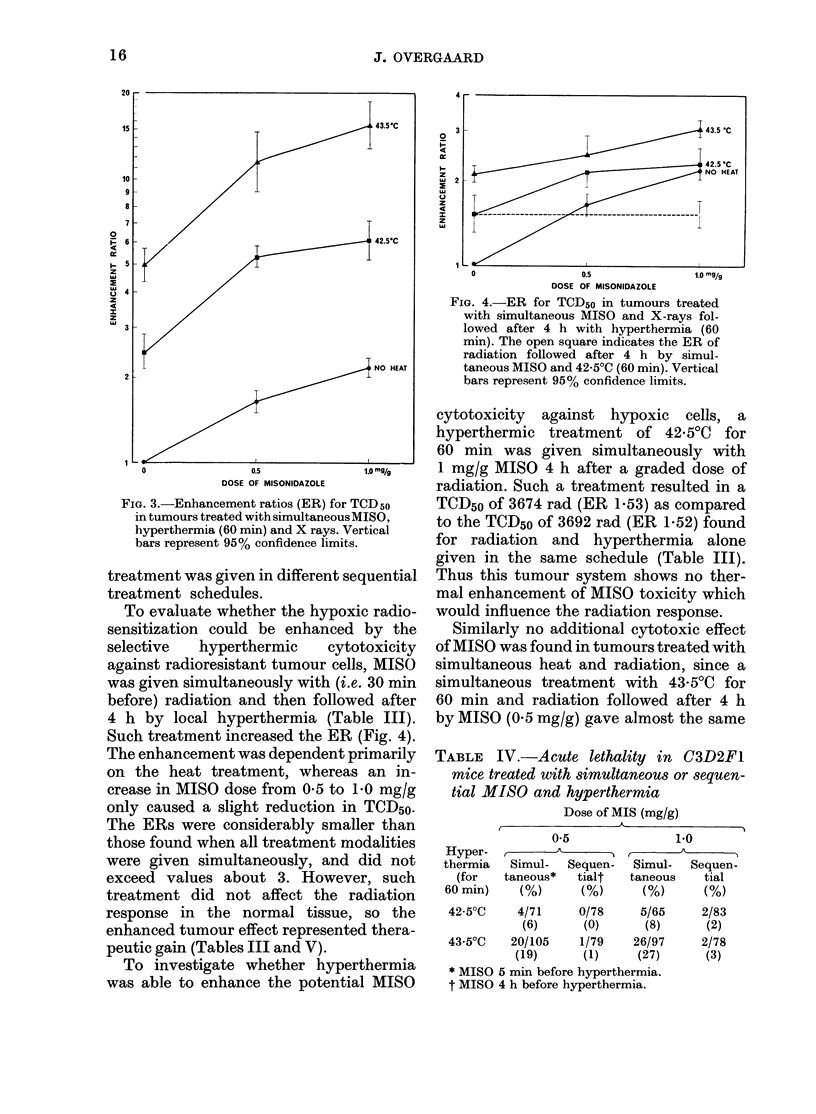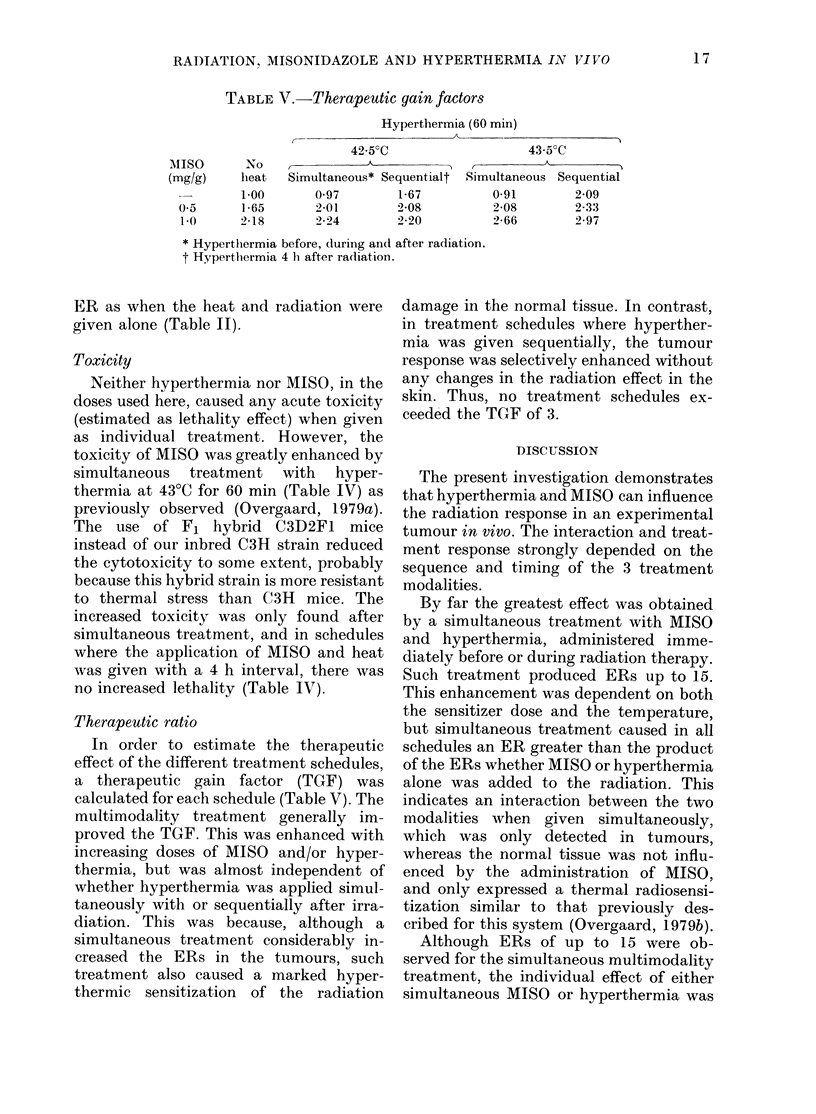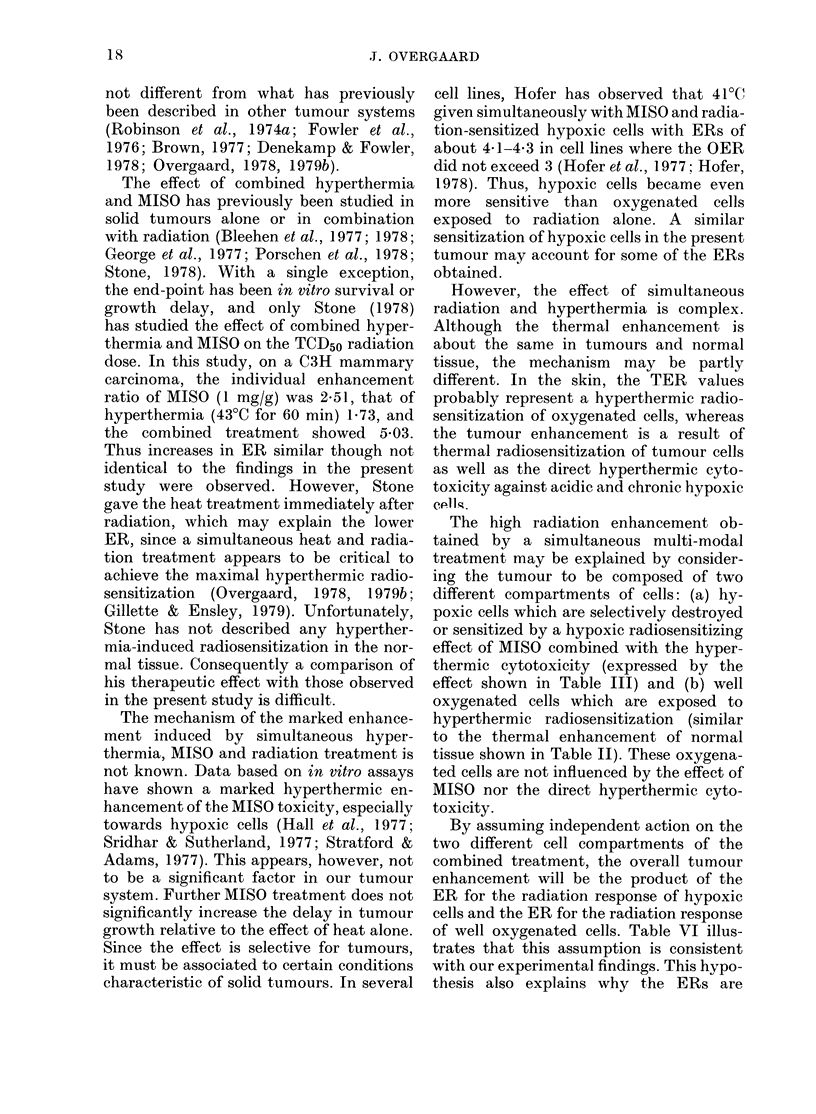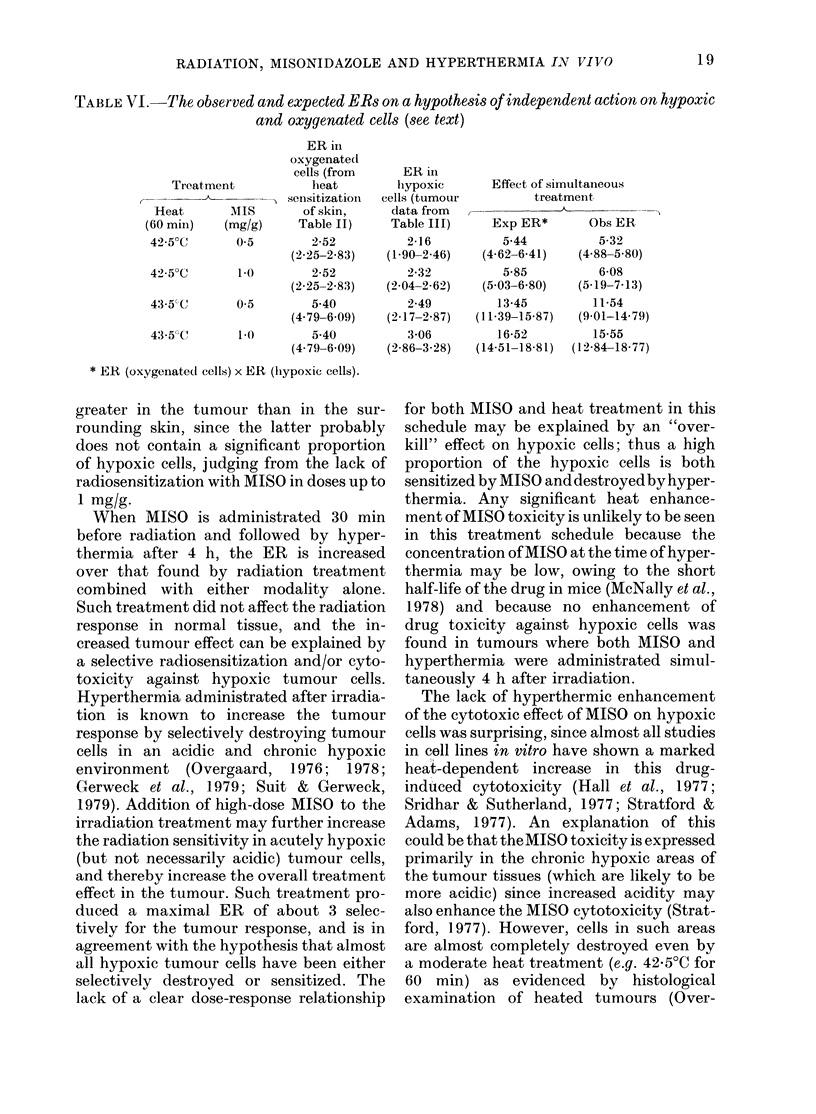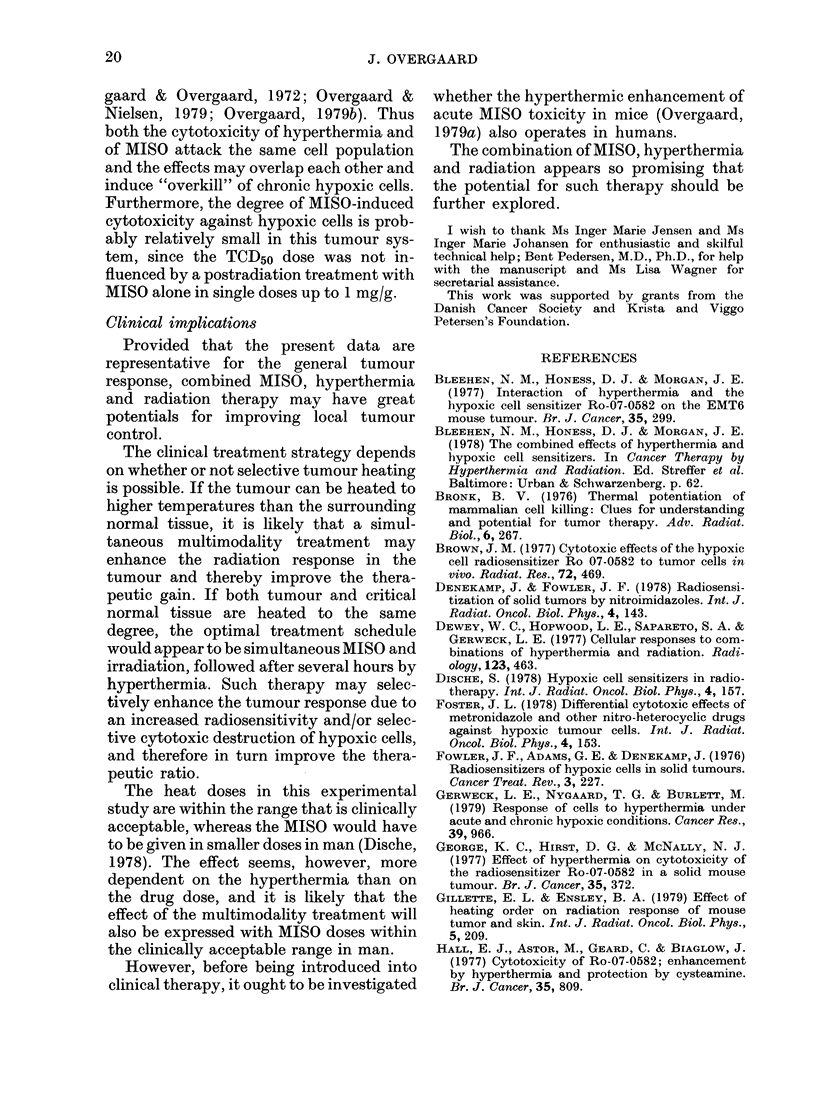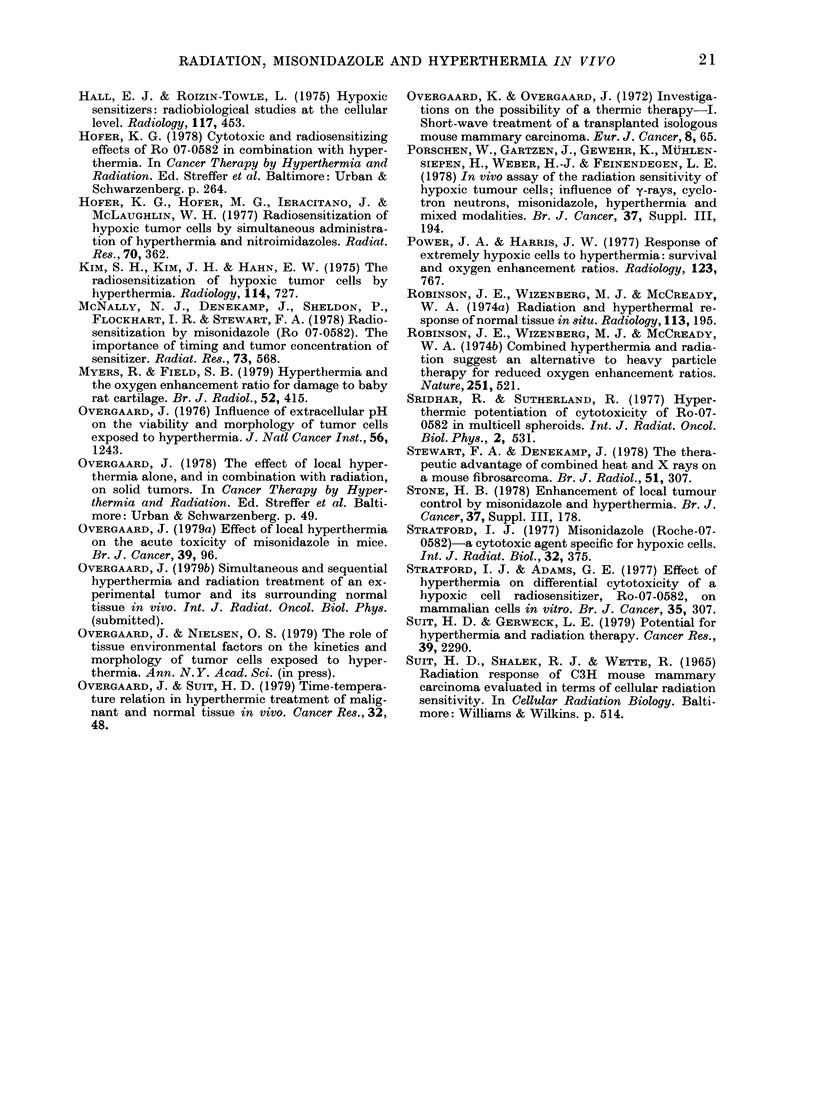# Effect of misonidazole and hyperthermia on the radiosensitivity of a C3H mouse mammary carcinoma and its surrounding normal tissue.

**DOI:** 10.1038/bjc.1980.2

**Published:** 1980-01

**Authors:** J. Overgaard

## Abstract

**Images:**


*Br. J. Cancer* (1980) **41**, 10

### EFFECT OF MISONIDAZOLE AND HYPERTHERMIA ON THE RADIOSENSITIVITY OF A C3H MOUSE MAMMARY CARCINOMA AND ITS SURROUNDING NORMAL TISSUE

J. OVERGAARD

*From the Institute of Cancer Research and the Department of Radiotherapy and Oncology, Radiumstationen, DK-8000 Aarhus C, Denmark*

Received 18 July 1979 Accepted 5 September 1979

**Summary.—Both misonidazole (MISO) and hyperthermia are known to enhance the radiation response of hypoxic cells, and to be selectively cytotoxic against cells in a hypoxic and acidic environment. The ability of these conditions to modify the effect of irradiation and their individual relationship was studied in a C3H mammary carcinoma and its surrounding skin.**

**Simultaneous treatment with MISO, hyperthermia and radiation increased the radiation effect, with enhancement ratios (ER) of up to about 15 (1 mg/g MISO and 43·5°C for 60 min.). However, such treatment also caused a smaller hyperthermic radiosensitization of the normal tissue, so that the therapeutic ratio was only increased by a factor of about 3 compared to radiation alone.**

**Simultaneous MISO and radiation followed by hyperthermia 4 h later gave a moderate enhancement, with ER up to 3 in the tumour, but with no enhancement of the normal tissue, so that there is a similar 3-fold increase in therapeutic gain.**

**The mechanism by which MISO and hyperthermia enhanced the radiation response may be explained as an independent action of the hypoxic radiosensitization of MISO and the selective hyperthermic cytotoxicity against acidic and chronic hypoxic cells; simultaneous hyperthermia added a further heat-induced general radiosensitization. Surprisingly, no MISO cytotoxicity could be detected in this tumour system, with or without simultaneous hyperthermia.**

**The results indicate that in the proper treatment schedule, MISO may be a valuable addition to a combined hyperthermia and radiation treatment.**

MISONIDAZOLE (MISO) and hyperthermia have a number of common features which make them potentially valuable in combined treatment with radiation for local tumour control.

Hyperthermia has been shown to sensitize to the effect of radiation. This occurs by several mechanisms including: direct increased cellular radiosensitivity, decreased accumulation of sublethal damage, and sensitization of cells in radioresistant phases of the cell cycle (Bronk, 1976; Dewey *et al.*, 1977). Furthermore, heat may sensitize hypoxic cells more than well oxygenated cells, thus causing a decreased oxygen enhancement ratio (Robinson *et al.*, 1974*a*,*b*; Kim *et al.*, 1975). However, the data on this special effect on hypoxic cells are ambiguous (Power & Harris, 1977; Myers & Field, 1979). The heat-induced radiosensitization is strongly dependent on the time of application of the two modalities. In general, optimal sensitization is obtained by simultaneous treatment, any interval between the two components tending to reduce the sensitization effect (Stewart & Denekamp, 1978; Overgaard, 1979*b*). If hyperthermia is given more than 4 h after radiation, the direct radiosensitizing effect is lost. When studied in normal tissues and tumours *in vivo*, the radiosensitizing effect of hyperthermia is approximately similar, and it is doubtful whether a simultaneous treatment would improve the therapeutic ratio (Gillette & Ensley, 1979; Overgaard, 1979*b*).

Besides its ability to act as a radiosensitizer, heat also has a direct cytotoxic effect, and may control experimental tumours with an acceptable degree of normal-tissue damage (Overgaard & Overgaard, 1972; Overgaard, 1978; Overgaard & Suit, 1979). This cytotoxicity is strongly enhanced by certain environment factors, and moderate hyperthermia is able to destroy almost selectively cells in areas of chronic hypoxia, acidity and insufficient nutrition (typical of large areas of solid tumours) (Overgaard, 1976, 1978; Gerweck *et al.*, 1979). The fact that cells in such an environment are also the most radioresistant may indirectly influence the response to combined heat–radiation treatment, since a smaller radiation dose may be adequate to control the remaining better-oxygenated peripheral tumour cells. In contrast to the hyperthermic radiosensitization, this cytotoxic effect shows no time relation to the radiation treatment (Overgaard, 1978, 1979*b*).

Misonidazole was originally introduced as a drug which sensitizes hypoxic cells for radiation (Fowler *et al.*, 1976; Denekamp & Fowler, 1978). This sensitization occurs only in hypoxic cells, and there is no influence on the radiation response of cells situated in a well oxygenated environment such as in most normal tissues.

More recent studies have furthermore shown that under hypoxia MISO may also exhibit a direct cytotoxic effect (Hall & Roizin-Towle, 1975; Fowler *et al.*, 1976; Brown, 1977; Foster, 1978). This effect resembles that of hyperthermia in that increased acidity also increases the cytotoxicity of MISO against hypoxic cells (Stratford, 1977). Both the radiosensitization of hypoxic cells and the cytotoxicity are dose-dependent; the radiosensitization generally occurs at lower doses than those causing measurable cytotoxicity effects in experimental solid tumours (Fowler *et al.*, 1976).

Not only are both modalities similar in their effective mechanisms but hyperthermia itself may also enhance the cytotoxicity of MISO (Hall *et al.*, 1977; Stratford & Adams, 1977; Bleehen *et al.*, 1978). However, detailed studies on these interactions are sparse. In particular, data on the effect in solid tumours are lacking. The present experiments were therefore undertaken to evaluate the relative influence of the radiosensitizing and cytotoxic effects of MISO and hyperthermia in a solid tumour and its surrounding tissue, in order to obtain an optimal therapeutic effect.

### MATERIAL AND METHODS

### *Animal tumour system*

Ten–12-week-old male and female C3D2F1/Bom (C3H/Tif♀ × DBA/2♂) mice were used. The animals were challenged with a spontaneously arisen C3H/Tif mammary carcinoma, which was propagated by serial transplantation. Tumour material for inoculum was obtained by sterile dissection of large flank tumours. Macroscopically viable tumour tissue was minced with a pair of scissors, and 5–10 $\mu$l of this minced tumour was injected into the foot on the right hind limb of the experimental animals. The transplant take was over 95%.

### *Treatment*

Treatment was given to tumours with a volume of ~200 mm$^3$ as determined by the formula $D_1 \times D_2 \times D_3 \times \pi/6$ where the Ds represent 3 orthogonal diameters. This treatment size was normally obtained about 14 days after inoculation. All treatments were given to unanaesthetized animals which were placed in a lucite jig with the tumour-bearing leg loosely fixed with tape without impairing the blood flow to the foot (Fig. 1).

*Hyperthermia*.—Local hyperthermia was administered with the tumour-bearing leg immersed in a circulating water bath (Heto type TE 623 or T 643) stabilized to ±0·02°C of the adjusted temperature. The water bath was covered with a lucite plate with holes allowing immersion into the water of the tumour-bearing leg. Previous measurements of intratumoural temperature have shown stabilization within a few minutes to approximately 0·2°C below the water-bath tempera-

FIG. 1.—Lucite jig for radiation and/or hyperthermic treatment. The unanaesthetized mouse is placed in the jig and the tumour-bearing leg is loosely taped to the plate, allowing immersion in the water bath.

ture (Overgaard & Suit, 1979; Overgaard, 1979*b*). The temperature of the water bath was therefore adjusted to 0·2°C above the desired tumour temperature. All further temperature references in this paper are to the intratumoral temperature. Temperature measurements were calibrated against a certified precision mercury thermometer. In all experiments the heating time was 60 min. For radiation given simultaneously with hyperthermia the tumours were radiated in the middle of the 1 h hyperthermic period. Sequential radiation and hyperthermia was performed by starting the hyperthermic treatment 4 h after completion of the radiation.

*Irradiation.*—Tumours were treated with graded single doses of radiation to produce dose-response data. The treatment was given with a conventional clinical X-ray machine with a dose rate of 190 rad/min (factors: 250 kV, 15 mA, 2mm Al filtration, 1·1mm Cu HVL). The unanaesthetized animals were placed in lucite jigs and radiated with the tumours immersed in a water bath and with ~5 cm of water between the X-ray source and the tumour, to secure the homogeneity of the radiation dose (Fig. 2). The remaining part of the animals was shielded with 4mm lead. For radiation given simultaneously with hyperthermia, the water bath was heated to a desired temperature. For all other radiations, the water bath had room temperature.

*Misonidazole.*—The drug was obtained through Roche Ltd, Copenhagen (by courtesy of Rud Hammer Jensen). It was dissolved in isotonic saline to a concentration of 20 mg/ml. This solution was injected i.p. into non-anaesthetized mice 30 min before the start of the irradiation. For treatments given simultaneously with hyperthermia, the drug was injected 5 min before the hyperthermic treatment, and radiation was then started after an additional 25 min. In experiments analysing the cytotoxicity of MISO, the drug was given either immediately or 4 h after irradiation.

### *Evaluation of results*

The animals were followed up with intervals of at least one week up to 120 days after treatment.

The response to treatment was measured as the radiation dose which would on the average be expected to control 50% of the treated tumours ($TCD_{50}$) at 120 days. The response of the normal tissue was determined as the radiation dose required to achieve a

FIG. 2.—Experimental set-up for combined hyperthermia and radiation treatment. The mice are placed with the tumour-bearing leg in a water bath and irradiated with a 250kV X-ray machine. During treatment the body of the mouse is shielded with 4 mm of lead (not shown). For simultaneous hyperthermia and radiation the water was heated to the desired temperature. Otherwise the radiation was given with water bath at room temperature.

full moist desquamation of the irradiated limb within 30 days in 50% of the animals ($DD_{50}$). The $TCD_{50}$ and $DD_{50}$ values were computed by logit analysis (Suit *et al.*, 1965).

The effect on the radiation response of an additional treatment was calculated as the "enhancement ratio" (ER) which is the radiation dose required to obtain a given end-point ($TCD_{50}$ or $DD_{50}$) with radiation alone relative to the radiation dose needed to obtain the same response with combined treatment.

Based on the ERs obtained in a given treatment schedule, a "therapeutic gain factor" (TGF) was calculated as the ER for the tumour relative to the ER for the normal tissue. This therapeutic gain factor was the ultimate objective of the study.

### RESULTS

### *Effect of misonidazole*

Administration of MISO 30 min before irradiation caused a significant decrease in the radiation $TCD_{50}$ (Table I). The effect depended on the drug dose, yielding ERs of 1·65 and 2·18 for doses of 0·5 mg/g and 1·0 mg/g MISO, respectively. This enhancement was obtained without altering the radiosensitivity of the surrounding skin, and therefore represented a similar improvement of the therapeutic effect. Single doses of MISO up to 1 mg/g *after* irradiation did not alter the $TCD_{50}$ significantly. Thus, in the present tumour MISO in single doses showed hypoxic radiosensitization without direct cytotoxicity against hypoxic cells.

### *Effect of hyperthermia*

As previously reported, the effect of hyperthermia depended on the sequence and interval between radiation and heat (Overgaard, 1979*b*). Simultaneous treatment produced the greatest thermal en-

TABLE I.—*Effect of misonidazole on the radiation response of a C3H mammary carcinoma*

| | Dose of MISO (mg/g) | No. of mice | $TCD_{50}$ (rad) | ER† |
|---|---|---|---|---|
| Radiation alone (control) | — | 248 | 5622 (5450–5787)* | — |
| MISO 30 min *before* radiation | 0·5 | 43 | 3415 (3040–3820) | 1·65 (1·50–1·80) |
| | 1·0 | 49 | 2574 (2349–2808) | 2·18 (2·03–2·35) |
| MISO immediately *after* radiation | 0·5 | 42 | 5564 (5237–5909) | 1·01 (0·96–1·06) |
| | 1·0 | 42 | 5528 (5037–6066) | 1·02 (0·95–1·08) |
| MISO 4 h *after* radiation | 0·5 | 43 | 5783 (5453–6136) | 0·97 (0·92–1·02) |

* In brackets 95% confidence limits.

† Enhancement ratio (ER) $= \frac{TCD_{50}\ \text{radiation alone}}{TCD_{50}\ \text{combined treatment}}$.

TABLE II.—*Effect of simultaneous MISO and/or simultaneous hyperthermia on the radiation response in a C3H mammary carcinoma and its surrounding skin*

| Treatment | | | Tumour response | | Skin response | | |
|---|---|---|---|---|---|---|---|
| MISO (30 min before radiation) | Hyperthermia 60 min (radiation during heating) | No. of mice | $TCD_{50}$ (rad) | ER† | $DD_{50}$ (rad) | ER | Therapeutic gain factor‡ (TGF) |
| Control | | 248 | 5622 (5450–5787)* | — | 2664 (2464–2882) | — | — |
| — | 42·5°C | 78 | 2299 (1913–2750) | 2·45 (2·15–2·83) | 1054 (947–1140) | 2·52 (2·25–2·83) | 0·97 |
| 0·5 mg/g | 42·5°C | 67 | 1056 (881–1266) | 5·32 (4·88–5·80) | 1009 (841–1210) | 2·64 (2·36–2·95) | 2·01 |
| 1·0 mg/g | 42·5°C | 60 | 924 (716–1192) | 6·08 (5·19–7·13) | 978 (743–1287) | 2·72 (2·21–3·35) | 2·24 |
| — | 43·5°C | 70 | 1146 (942–1396) | 4·91 (4·26–5·64) | 493 (431–562) | 5·40 (4·79–6·09) | 0·91 |
| 0·5 mg/g | 43·5°C | 85 | 487 (283–837) | 11·54 (9·01–14·79) | 483 (293–783) | 5·55 (4·75–6·48) | 2·08 |
| 0·5 mg/g (4 h after heat and radiation) | 43·5°C | 35 | 1014 (758–1264) | 5·54 (4·51–6·82) | 500 (222–1107) | 5·33 (3·65–7·76) | 1·04 |
| 1·0 mg/g | 43·5°C | 71 | 362 (278–471) | 15·55 (12·84–18·77) | 456 (335–625) | 5·84 (4·75–7·17) | 2·66 |

* In brackets 95% confidence limits.

† Enhancement ratio (ER) $= \frac{\text{Response dose to radiation}}{\text{Response dose to combined treatment}}$.

‡ TGF $= \frac{\text{ER tumour}}{\text{ER skin}}$.

hancement, but to the same degree in both tumour and normal tissue, and a therapeutic gain was therefore doubtful (Table II). On the other hand, selective tumour cytotoxicity was expressed if the hyperthermia was given 4 h *after* radiation. Such treatment reduces the $TCD_{50}$, but did not enhance the radiation response in the surrounding normal tissue (Table III). A sequential treatment thus improved the therapeutic gain. It is reasonable to assume that the effect of simultaneous hyperthermia and radiation treatment is mainly due to hyperthermic radiosensi-

TABLE III.—*Effect of simultaneous MISO and/or sequential hyperthermia on the radiation response in a C3H mammary carcinoma and its surrounding skin*

| Treatment: MISO (30 min before radiation) | Treatment: Hyperthermia (4 h for 60 min after radiation) | No. of mice | Tumour response: $TCD_{50}$ (rad) | Tumour response: ER† | Skin response: $DD_{50}$ (rad) | Skin response: ER‡ | TGF |
|---|---|---|---|---|---|---|---|
| Control | | 248 | 5622 (5450–5787)* | — | 2664 (2464–2882) | — | — |
| — | 42·5°C | 85 | 3692 (2838–4717) | 1·52 (1·31–1·77) | 2931 (2631–3266) | 0·91 (0·82–1·01) | 1·67 |
| 0·5 mg/g | 42·5°C | 78 | 2598 (1964–3436) | 2·16 (1·90–2·46) | 2568 (1720–3820) | 1·04 (0·94–1·14) | 2·08 |
| 1·0 mg/g | 42·5°C | 81 | 2434 (2045–2895) | 2·32 (2·04–2·62) | 2525 (2122–3006) | 1·05 (0·93–1·20) | 2·20 |
| 1·0 mg/g (4 h after radiation) | 42·5°C | 34 | 3674 (2890–4673) | 1·53 (1·37–1·71) | 2855 (2458–3315) | 0·93 (0·84–1·03) | 1·64 |
| — | 43·5°C | 83 | 2668 (2315–3073) | 2·12 (1·97–2·26) | 2641 (2253–3132) | 1·01 (0·91–1·12) | 2·09 |
| 0·5 mg/g | 43·5°C | 78 | 2255 (1857–2734) | 2·49 (2·17–2·87) | 2493 (1798–3456) | 1·07 (0·91–1·25) | 2·33 |
| 1·0 mg/g | 43·5°C | 76 | 1836 (1588–2122) | 3·06 (2·86–3·28) | 2575 (2240–2935) | 1·03 (0·92–1·21) | 2·97 |

* In brackets 95% confidence limits.

† Enhancement ratio (ER) $= \frac{\text{Response dose to radiation}}{\text{Response dose to combined treatment}}$.

tization, whereas the sequential treatment expresses selective hyperthermic cytotoxicity against radioresistant (acidic and chronic hypoxic) tumour cells.

*Effect of simultaneous hyperthermia and MISO*

The interaction between hyperthermia and MISO was first studied in a treatment schedule where the modalities were applied simultaneously (Table II). Such a treatment caused a dramatic increase in the ER of the radiation response in the tumours, ER of up to about 15. This effect was considerably more than additive. The increased ER was due to both the dose of MISO and the heat treatment with the latter as the most decisive factor (Fig. 3).

Although an additional treatment with 43·5°C for 60 min combined with 1 mg/g misonidazole caused a decreased $TCD_{50}$ from 5622 rad to 380 rad, radiation was still required to control the tumours. In fact, in this relatively heat-resistant tumour, hyperthermia induced only a minor reduction in growth delay, and simultaneous MISO and heat caused no significant delay in tumour growth when compared to tumours heated alone (data not shown).

A simultaneous multimodality treatment also increased the radiation response in normal tissue. This enhancement was similar to that after simultaneous heat and radiation treatment alone, so the addition of MISO only caused extra enhancement of the tumour response, which in turn increased the therapeutic ratio (Tables II and V).

*Effect of sequential multimodality treatment*

In order to investigate the relative importance of hypoxic radiosensitization and the direct hypoxic cytotoxicity, the

FIG. 3.—Enhancement ratios (ER) for $TCD_{50}$ in tumours treated with simultaneous MISO, hyperthermia (60 min) and X rays. Vertical bars represent 95% confidence limits.

FIG. 4.—ER for $TCD_{50}$ in tumours treated with simultaneous MISO and X-rays followed after 4 h with hyperthermia (60 min). The open square indicates the ER of radiation followed after 4 h by simultaneous MISO and 42·5°C (60 min). Vertical bars represent 95% confidence limits.

treatment was given in different sequential treatment schedules.

To evaluate whether the hypoxic radiosensitization could be enhanced by the selective hyperthermic cytotoxicity against radioresistant tumour cells, MISO was given simultaneously with (*i.e.* 30 min before) radiation and then followed after 4 h by local hyperthermia (Table III). Such treatment increased the ER (Fig. 4). The enhancement was dependent primarily on the heat treatment, whereas an increase in MISO dose from 0·5 to 1·0 mg/g only caused a slight reduction in $TCD_{50}$. The ERs were considerably smaller than those found when all treatment modalities were given simultaneously, and did not exceed values about 3. However, such treatment did not affect the radiation response in the normal tissue, so the enhanced tumour effect represented therapeutic gain (Tables III and V).

To investigate whether hyperthermia was able to enhance the potential MISO cytotoxicity against hypoxic cells, a hyperthermic treatment of 42·5°C for 60 min was given simultaneously with 1 mg/g MISO 4 h after a graded dose of radiation. Such a treatment resulted in a $TCD_{50}$ of 3674 rad (ER 1·53) as compared to the $TCD_{50}$ of 3692 rad (ER 1·52) found for radiation and hyperthermia alone given in the same schedule (Table III). Thus this tumour system shows no thermal enhancement of MISO toxicity which would influence the radiation response.

Similarly no additional cytotoxic effect of MISO was found in tumours treated with simultaneous heat and radiation, since a simultaneous treatment with 43·5°C for 60 min and radiation followed after 4 h by MISO (0·5 mg/g) gave almost the same

TABLE IV.—*Acute lethality in C3D2F1 mice treated with simultaneous or sequential MISO and hyperthermia*

| Hyperthermia (for 60 min) | Dose of MIS (mg/g) 0·5 Simultaneous* (%) | 0·5 Sequential† (%) | 1·0 Simultaneous (%) | 1·0 Sequential (%) |
|---|---|---|---|---|
| 42·5°C | 4/71 (6) | 0/78 (0) | 5/65 (8) | 2/83 (2) |
| 43·5°C | 20/105 (19) | 1/79 (1) | 26/97 (27) | 2/78 (3) |

* MISO 5 min before hyperthermia.
† MISO 4 h before hyperthermia.

TABLE V.—*Therapeutic gain factors*

| | | Hyperthermia (60 min) | | | |
|---|---|---|---|---|---|
| | | 42·5°C | | 43·5°C | |
| MISO (mg/g) | No heat | Simultaneous* | Sequential† | Simultaneous | Sequential |
| — | 1·00 | 0·97 | 1·67 | 0·91 | 2·09 |
| 0·5 | 1·65 | 2·01 | 2·08 | 2·08 | 2·33 |
| 1·0 | 2·18 | 2·24 | 2·20 | 2·66 | 2·97 |

* Hyperthermia before, during and after radiation.
† Hyperthermia 4 h after radiation.

ER as when the heat and radiation were given alone (Table II).

*Toxicity*

Neither hyperthermia nor MISO, in the doses used here, caused any acute toxicity (estimated as lethality effect) when given as individual treatment. However, the toxicity of MISO was greatly enhanced by simultaneous treatment with hyperthermia at 43°C for 60 min (Table IV) as previously observed (Overgaard, 1979*a*). The use of $F_1$ hybrid C3D2F1 mice instead of our inbred C3H strain reduced the cytotoxicity to some extent, probably because this hybrid strain is more resistant to thermal stress than C3H mice. The increased toxicity was only found after simultaneous treatment, and in schedules where the application of MISO and heat was given with a 4 h interval, there was no increased lethality (Table IV).

*Therapeutic ratio*

In order to estimate the therapeutic effect of the different treatment schedules, a therapeutic gain factor (TGF) was calculated for each schedule (Table V). The multimodality treatment generally improved the TGF. This was enhanced with increasing doses of MISO and/or hyperthermia, but was almost independent of whether hyperthermia was applied simultaneously with or sequentially after irradiation. This was because, although a simultaneous treatment considerably increased the ERs in the tumours, such treatment also caused a marked hyperthermic sensitization of the radiation damage in the normal tissue. In contrast, in treatment schedules where hyperthermia was given sequentially, the tumour response was selectively enhanced without any changes in the radiation effect in the skin. Thus, no treatment schedules exceeded the TGF of 3.

### DISCUSSION

The present investigation demonstrates that hyperthermia and MISO can influence the radiation response in an experimental tumour *in vivo*. The interaction and treatment response strongly depended on the sequence and timing of the 3 treatment modalities.

By far the greatest effect was obtained by a simultaneous treatment with MISO and hyperthermia, administered immediately before or during radiation therapy. Such treatment produced ERs up to 15. This enhancement was dependent on both the sensitizer dose and the temperature, but simultaneous treatment caused in all schedules an ER greater than the product of the ERs whether MISO or hyperthermia alone was added to the radiation. This indicates an interaction between the two modalities when given simultaneously, which was only detected in tumours, whereas the normal tissue was not influenced by the administration of MISO, and only expressed a thermal radiosensitization similar to that previously described for this system (Overgaard, 1979*b*).

Although ERs of up to 15 were observed for the simultaneous multimodality treatment, the individual effect of either simultaneous MISO or hyperthermia was not different from what has previously been described in other tumour systems (Robinson *et al.*, 1974*a*; Fowler *et al.*, 1976; Brown, 1977; Denekamp & Fowler, 1978; Overgaard, 1978, 1979*b*).

The effect of combined hyperthermia and MISO has previously been studied in solid tumours alone or in combination with radiation (Bleehen *et al.*, 1977; 1978; George *et al.*, 1977; Porschen *et al.*, 1978; Stone, 1978). With a single exception, the end-point has been *in vitro* survival or growth delay, and only Stone (1978) has studied the effect of combined hyperthermia and MISO on the $TCD_{50}$ radiation dose. In this study, on a C3H mammary carcinoma, the individual enhancement ratio of MISO (1 mg/g) was 2·51, that of hyperthermia (43°C for 60 min) 1·73, and the combined treatment showed 5·03. Thus increases in ER similar though not identical to the findings in the present study were observed. However, Stone gave the heat treatment immediately after radiation, which may explain the lower ER, since a simultaneous heat and radiation treatment appears to be critical to achieve the maximal hyperthermic radiosensitization (Overgaard, 1978, 1979*b*; Gillette & Ensley, 1979). Unfortunately, Stone has not described any hyperthermia-induced radiosensitization in the normal tissue. Consequently a comparison of his therapeutic effect with those observed in the present study is difficult.

The mechanism of the marked enhancement induced by simultaneous hyperthermia, MISO and radiation treatment is not known. Data based on *in vitro* assays have shown a marked hyperthermic enhancement of the MISO toxicity, especially towards hypoxic cells (Hall *et al.*, 1977; Sridhar & Sutherland, 1977; Stratford & Adams, 1977). This appears, however, not to be a significant factor in our tumour system. Further MISO treatment does not significantly increase the delay in tumour growth relative to the effect of heat alone. Since the effect is selective for tumours, it must be associated to certain conditions characteristic of solid tumours. In several cell lines, Hofer has observed that 41°C given simultaneously with MISO and radiation-sensitized hypoxic cells with ERs of about 4·1–4·3 in cell lines where the OER did not exceed 3 (Hofer *et al.*, 1977; Hofer, 1978). Thus, hypoxic cells became even more sensitive than oxygenated cells exposed to radiation alone. A similar sensitization of hypoxic cells in the present tumour may account for some of the ERs obtained.

However, the effect of simultaneous radiation and hyperthermia is complex. Although the thermal enhancement is about the same in tumours and normal tissue, the mechanism may be partly different. In the skin, the TER values probably represent a hyperthermic radiosensitization of oxygenated cells, whereas the tumour enhancement is a result of thermal radiosensitization of tumour cells as well as the direct hyperthermic cytotoxicity against acidic and chronic hypoxic cells.

The high radiation enhancement obtained by a simultaneous multi-modal treatment may be explained by considering the tumour to be composed of two different compartments of cells: (a) hypoxic cells which are selectively destroyed or sensitized by a hypoxic radiosensitizing effect of MISO combined with the hyperthermic cytotoxicity (expressed by the effect shown in Table III) and (b) well oxygenated cells which are exposed to hyperthermic radiosensitization (similar to the thermal enhancement of normal tissue shown in Table II). These oxygenated cells are not influenced by the effect of MISO nor the direct hyperthermic cytotoxicity.

By assuming independent action on the two different cell compartments of the combined treatment, the overall tumour enhancement will be the product of the ER for the radiation response of hypoxic cells and the ER for the radiation response of well oxygenated cells. Table VI illustrates that this assumption is consistent with our experimental findings. This hypothesis also explains why the ERs are

TABLE VI.—*The observed and expected ERs on a hypothesis of independent action on hypoxic and oxygenated cells (see text)*

| Treatment | | ER in oxygenated cells (from heat sensitization of skin, Table II) | ER in hypoxic cells (tumour data from Table III) | Effect of simultaneous treatment | |
|---|---|---|---|---|---|
| Heat (60 min) | MIS (mg/g) | | | Exp ER* | Obs ER |
| 42·5°C | 0·5 | 2·52 (2·25–2·83) | 2·16 (1·90–2·46) | 5·44 (4·62–6·41) | 5·32 (4·88–5·80) |
| 42·5°C | 1·0 | 2·52 (2·25–2·83) | 2·32 (2·04–2·62) | 5·85 (5·03–6·80) | 6·08 (5·19–7·13) |
| 43·5°C | 0·5 | 5·40 (4·79–6·09) | 2·49 (2·17–2·87) | 13·45 (11·39–15·87) | 11·54 (9·01–14·79) |
| 43·5°C | 1·0 | 5·40 (4·79–6·09) | 3·06 (2·86–3·28) | 16·52 (14·51–18·81) | 15·55 (12·84–18·77) |

* ER (oxygenated cells) × ER (hypoxic cells).

greater in the tumour than in the surrounding skin, since the latter probably does not contain a significant proportion of hypoxic cells, judging from the lack of radiosensitization with MISO in doses up to 1 mg/g.

When MISO is administrated 30 min before radiation and followed by hyperthermia after 4 h, the ER is increased over that found by radiation treatment combined with either modality alone. Such treatment did not affect the radiation response in normal tissue, and the increased tumour effect can be explained by a selective radiosensitization and/or cytotoxicity against hypoxic tumour cells. Hyperthermia administrated after irradiation is known to increase the tumour response by selectively destroying tumour cells in an acidic and chronic hypoxic environment (Overgaard, 1976; 1978; Gerweck *et al.*, 1979; Suit & Gerweck, 1979). Addition of high-dose MISO to the irradiation treatment may further increase the radiation sensitivity in acutely hypoxic (but not necessarily acidic) tumour cells, and thereby increase the overall treatment effect in the tumour. Such treatment produced a maximal ER of about 3 selectively for the tumour response, and is in agreement with the hypothesis that almost all hypoxic tumour cells have been either selectively destroyed or sensitized. The lack of a clear dose-response relationship for both MISO and heat treatment in this schedule may be explained by an "overkill" effect on hypoxic cells; thus a high proportion of the hypoxic cells is both sensitized by MISO and destroyed by hyperthermia. Any significant heat enhancement of MISO toxicity is unlikely to be seen in this treatment schedule because the concentration of MISO at the time of hyperthermia may be low, owing to the short half-life of the drug in mice (McNally *et al.*, 1978) and because no enhancement of drug toxicity against hypoxic cells was found in tumours where both MISO and hyperthermia were administrated simultaneously 4 h after irradiation.

The lack of hyperthermic enhancement of the cytotoxic effect of MISO on hypoxic cells was surprising, since almost all studies in cell lines *in vitro* have shown a marked heat-dependent increase in this drug-induced cytotoxicity (Hall *et al.*, 1977; Sridhar & Sutherland, 1977; Stratford & Adams, 1977). An explanation of this could be that the MISO toxicity is expressed primarily in the chronic hypoxic areas of the tumour tissues (which are likely to be more acidic) since increased acidity may also enhance the MISO cytotoxicity (Stratford, 1977). However, cells in such areas are almost completely destroyed even by a moderate heat treatment (*e.g.* 42·5°C for 60 min) as evidenced by histological examination of heated tumours (Overgaard & Overgaard, 1972; Overgaard & Nielsen, 1979; Overgaard, 1979*b*). Thus both the cytotoxicity of hyperthermia and of MISO attack the same cell population and the effects may overlap each other and induce "overkill" of chronic hypoxic cells. Furthermore, the degree of MISO-induced cytotoxicity against hypoxic cells is probably relatively small in this tumour system, since the $TCD_{50}$ dose was not influenced by a postradiation treatment with MISO alone in single doses up to 1 mg/g.

*Clinical implications*

Provided that the present data are representative for the general tumour response, combined MISO, hyperthermia and radiation therapy may have great potentials for improving local tumour control.

The clinical treatment strategy depends on whether or not selective tumour heating is possible. If the tumour can be heated to higher temperatures than the surrounding normal tissue, it is likely that a simultaneous multimodality treatment may enhance the radiation response in the tumour and thereby improve the therapeutic gain. If both tumour and critical normal tissue are heated to the same degree, the optimal treatment schedule would appear to be simultaneous MISO and irradiation, followed after several hours by hyperthermia. Such therapy may selectively enhance the tumour response due to an increased radiosensitivity and/or selective cytotoxic destruction of hypoxic cells, and therefore in turn improve the therapeutic ratio.

The heat doses in this experimental study are within the range that is clinically acceptable, whereas the MISO would have to be given in smaller doses in man (Dische, 1978). The effect seems, however, more dependent on the hyperthermia than on the drug dose, and it is likely that the effect of the multimodality treatment will also be expressed with MISO doses within the clinically acceptable range in man.

However, before being introduced into clinical therapy, it ought to be investigated whether the hyperthermic enhancement of acute MISO toxicity in mice (Overgaard, 1979*a*) also operates in humans.

The combination of MISO, hyperthermia and radiation appears so promising that the potential for such therapy should be further explored.

I wish to thank Ms Inger Marie Jensen and Ms Inger Marie Johansen for enthusiastic and skilful technical help; Bent Pedersen, M.D., Ph.D., for help with the manuscript and Ms Lisa Wagner for secretarial assistance.

This work was supported by grants from the Danish Cancer Society and Krista and Viggo Petersen's Foundation.

REFERENCES

Bleehen, N. M., Honess, D. J. & Morgan, J. E. (1977) Interaction of hyperthermia and the hypoxic cell sensitizer Ro-07-0582 on the EMT6 mouse tumour. *Br. J. Cancer*, **35**, 299.

Bleehen, N. M., Honess, D. J. & Morgan, J. E. (1978) The combined effects of hyperthermia and hypoxic cell sensitizers. In *Cancer Therapy by Hyperthermia and Radiation*. Ed. Streffer *et al.* Baltimore: Urban & Schwarzenberg. p. 62.

Bronk, B. V. (1976) Thermal potentiation of mammalian cell killing: Clues for understanding and potential for tumor therapy. *Adv. Radiat. Biol.*, **6**, 267.

Brown, J. M. (1977) Cytotoxic effects of the hypoxic cell radiosensitizer Ro 07-0582 to tumor cells *in vivo*. *Radiat. Res.*, **72**, 469.

Denekamp, J. & Fowler, J. F. (1978) Radiosensitization of solid tumors by nitroimidazoles. *Int. J. Radiat. Oncol. Biol. Phys.*, **4**, 143.

Dewey, W. C., Hopwood, L. E., Sapareto, S. A. & Gerweck, L. E. (1977) Cellular responses to combinations of hyperthermia and radiation. *Radiology*, **123**, 463.

Dische, S. (1978) Hypoxic cell sensitizers in radiotherapy. *Int. J. Radiat. Oncol. Biol. Phys.*, **4**, 157.

Foster, J. L. (1978) Differential cytotoxic effects of metronidazole and other nitro-heterocyclic drugs against hypoxic tumour cells. *Int. J. Radiat. Oncol. Biol. Phys.*, **4**, 153.

Fowler, J. F., Adams, G. E. & Denekamp, J. (1976) Radiosensitizers of hypoxic cells in solid tumours. *Cancer Treat. Rev.*, **3**, 227.

Gerweck, L. E., Nygaard, T. G. & Burlett, M. (1979) Response of cells to hyperthermia under acute and chronic hypoxic conditions. *Cancer Res.*, **39**, 966.

George, K. C., Hirst, D. G. & McNally, N. J. (1977) Effect of hyperthermia on cytotoxicity of the radiosensitizer Ro-07-0582 in a solid mouse tumour. *Br. J. Cancer*, **35**, 372.

Gillette, E. L. & Ensley, B. A. (1979) Effect of heating order on radiation response of mouse tumor and skin. *Int. J. Radiat. Oncol. Biol. Phys.*, **5**, 209.

Hall, E. J., Astor, M., Geard, C. & Biaglow, J. (1977) Cytotoxicity of Ro-07-0582; enhancement by hyperthermia and protection by cysteamine. *Br. J. Cancer*, **35**, 809.

HALL, E. J. & ROIZIN-TOWLE, L. (1975) Hypoxic sensitizers: radiobiological studies at the cellular level. *Radiology*, **117**, 453.

HOFER, K. G. (1978) Cytotoxic and radiosensitizing effects of Ro 07-0582 in combination with hyperthermia. In *Cancer Therapy by Hyperthermia and Radiation*. Ed. Streffer *et al.* Baltimore: Urban & Schwarzenberg. p. 264.

HOFER, K. G., HOFER, M. G., IERACITANO, J. & MCLAUGHLIN, W. H. (1977) Radiosensitization of hypoxic tumor cells by simultaneous administration of hyperthermia and nitroimidazoles. *Radiat. Res.*, **70**, 362.

KIM, S. H., KIM, J. H. & HAHN, E. W. (1975) The radiosensitization of hypoxic tumor cells by hyperthermia. *Radiology*, **114**, 727.

MCNALLY, N. J., DENEKAMP, J., SHELDON, P., FLOCKHART, I. R. & STEWART, F. A. (1978) Radiosensitization by misonidazole (Ro 07-0582). The importance of timing and tumor concentration of sensitizer. *Radiat. Res.*, **73**, 568.

MYERS, R. & FIELD, S. B. (1979) Hyperthermia and the oxygen enhancement ratio for damage to baby rat cartilage. *Br. J. Radiol.*, **52**, 415.

OVERGAARD, J. (1976) Influence of extracellular pH on the viability and morphology of tumor cells exposed to hyperthermia. *J. Natl Cancer Inst.*, **56**, 1243.

OVERGAARD, J. (1978) The effect of local hyperthermia alone, and in combination with radiation, on solid tumors. In *Cancer Therapy by Hyperthermia and Radiation*. Ed. Streffer *et al.* Baltimore: Urban & Schwarzenberg. p. 49.

OVERGAARD, J. (1979*a*) Effect of local hyperthermia on the acute toxicity of misonidazole in mice. *Br. J. Cancer*, **39**, 96.

OVERGAARD, J. (1979*b*) Simultaneous and sequential hyperthermia and radiation treatment of an experimental tumor and its surrounding normal tissue *in vivo*. *Int. J. Radiat. Oncol. Biol. Phys.* (submitted).

OVERGAARD, J. & NIELSEN, O. S. (1979) The role of tissue environmental factors on the kinetics and morphology of tumor cells exposed to hyperthermia. *Ann. N.Y. Acad. Sci.* (in press).

OVERGAARD, J. & SUIT, H. D. (1979) Time-temperature relation in hyperthermic treatment of malignant and normal tissue *in vivo*. *Cancer Res.*, **32**, **48**.

OVERGAARD, K. & OVERGAARD, J. (1972) Investigations on the possibility of a thermic therapy—I. Short-wave treatment of a transplanted isologous mouse mammary carcinoma. *Eur. J. Cancer*, **8**, 65.

PORSCHEN, W., GARTZEN, J., GEWEHR, K., MÜHLENSIEPEN, H., WEBER, H.-J. & FEINENDEGEN, L. E. (1978) *In vivo* assay of the radiation sensitivity of hypoxic tumour cells; influence of γ-rays, cyclotron neutrons, misonidazole, hyperthermia and mixed modalities. *Br. J. Cancer*, **37**, Suppl. III, 194.

POWER, J. A. & HARRIS, J. W. (1977) Response of extremely hypoxic cells to hyperthermia: survival and oxygen enhancement ratios. *Radiology*, **123**, 767.

ROBINSON, J. E., WIZENBERG, M. J. & MCCREADY, W. A. (1974*a*) Radiation and hyperthermal response of normal tissue *in situ*. *Radiology*, **113**, 195.

ROBINSON, J. E., WIZENBERG, M. J. & MCCREADY, W. A. (1974*b*) Combined hyperthermia and radiation suggest an alternative to heavy particle therapy for reduced oxygen enhancement ratios. *Nature*, **251**, 521.

SRIDHAR, R. & SUTHERLAND, R. (1977) Hyperthermic potentiation of cytotoxicity of Ro-07-0582 in multicell spheroids. *Int. J. Radiat. Oncol. Biol. Phys.*, **2**, 531.

STEWART, F. A. & DENEKAMP, J. (1978) The therapeutic advantage of combined heat and X rays on a mouse fibrosarcoma. *Br. J. Radiol.*, **51**, 307.

STONE, H. B. (1978) Enhancement of local tumour control by misonidazole and hyperthermia. *Br. J. Cancer*, **37**, Suppl. III, 178.

STRATFORD, I. J. (1977) Misonidazole (Roche-07-0582)—a cytotoxic agent specific for hypoxic cells. *Int. J. Radiat. Biol.*, **32**, 375.

STRATFORD, I. J. & ADAMS, G. E. (1977) Effect of hyperthermia on differential cytotoxicity of a hypoxic cell radiosensitizer, Ro-07-0582, on mammalian cells *in vitro*. *Br. J. Cancer*, **35**, 307.

SUIT, H. D. & GERWECK, L. E. (1979) Potential for hyperthermia and radiation therapy. *Cancer Res.*, **39**, 2290.

SUIT, H. D., SHALEK, R. J. & WETTE, R. (1965) Radiation response of C3H mouse mammary carcinoma evaluated in terms of cellular radiation sensitivity. In *Cellular Radiation Biology*. Baltimore: Williams & Wilkins. p. 514.